# Case report: Antenatal diagnosis of congenital high airway obstruction syndrome – laryngeal atresia

**DOI:** 10.4103/0971-3026.43843

**Published:** 2008-11

**Authors:** Mukesh Kumar Garg

**Affiliations:** Department of Radiology, Geetanjali Medical College and Hospital, Udaipur, (Rajasthan), India

**Keywords:** Congenital high airway obstruction syndrome, laryngeal atresia

## Abstract

Congenital high airway obstruction syndrome (CHAOS) is a near fatal condition of multifactorial inheritence, in which the fetus has a dilated trachea, enlarged echogenic lungs, an inverted or flattened diaphagram, and ascites. A case of CHAOS, diagnosed antenatally on USG at 28 weeks of gestation, is being reported here.

Congenital high airway obstruction syndrome (CHAOS) is a condition in which the fetus has hyperinflated, enlarged, and highly echogenic lungs; an inverted or flattened diaphragm; a dilated tracheobronchial tree; and ascites. It occurs as a result of congenital obstruction of the fetal airway secondary to laryngeal atresia, tracheal atresia, or a laryngeal cyst.[1–2] The disease is generally incompatible with life and, therefore, antenatal USG diagnosis is desirable. I would like to report a case where antenatal diagnosis was possible on USG at 28 weeks' gestation.

## Case Report

A 20-year-old multiparous (gravida 2) woman at 28 weeks' gestation was referred for a fetal well-being examination. There was no history of consanguinity and the family history was unremarkable. Her previous pregnancy had been uneventful.

**Figure 1:**
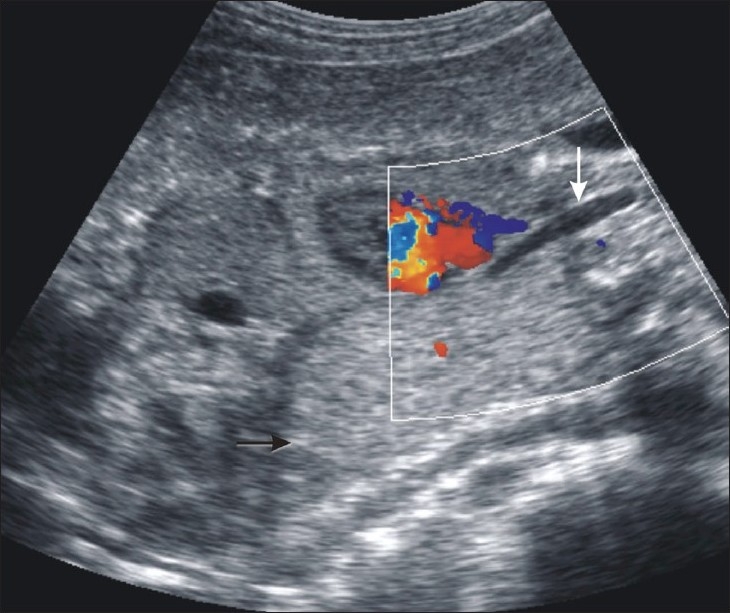
USG of the fetus in the coronal plane, at the level of the thorax shows a dilated trachea (white arrow). The black arrow points towards the flattened diaphragm

**Figure 2:**
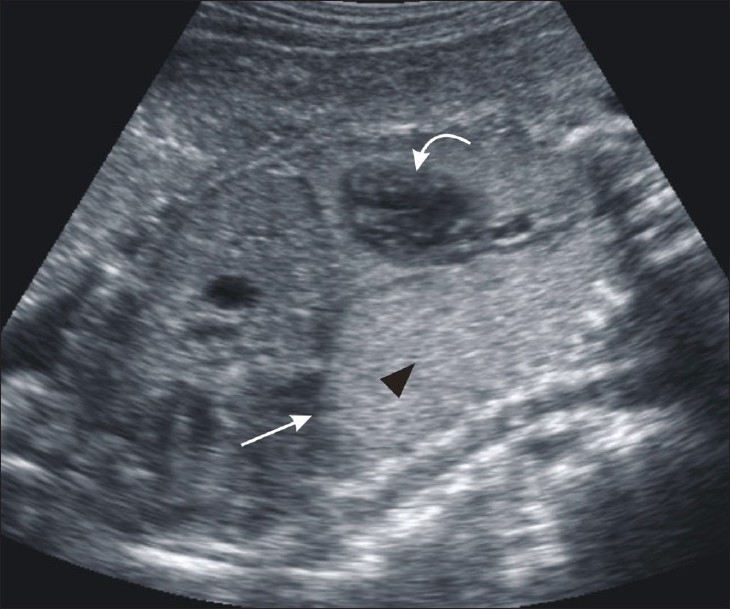
USG of the fetus in the coronal plane shows enlarged hyperechoiclungs (arrowhead) with flattened diaphragms (arrow) and a small heart (curved arrow)

USG showed a dilated trachea [Figure 1], enlarged hyperechoic lungs, inferiorly displaced and flattened diaphragms [Figures 1 and 2], minimal fetal ascites, excessive amniotic fluid volume (amniotic fluid index: 20 cm), and a small heart because of compression by the obstructed lungs [Figure 2]. These findings were diagnostic of CHAOS. We discussed the possible unfavorable outcome of the pregnancy with the parents who chose not to terminate the pregnancy because of religious reasons.

## Discussion

Laryngeal atresia is a rare congenital malformation and is usually fatal. The malformation is caused by nondevelopment of the 6^th^branchial arch during normal embryological development.[3] Smith and Bain[3] have classified laryngeal atresia into three types: type 1, in which there is complete atresia of the larynx with midline fusion of the arytenoid cartilages and intrinsic muscles; type 2, in which there is infraglottic obstruction that is characterized by a dome-shaped cricoid cartilage obstructing the lumen; and type 3, in which there is occlusion of the anterior fibrous membrane and fusion of the arytenoid cartilages at the level of the vocal processes.[3]

Association of laryngeal atresia with partial trisomy 9 and 16, resulting in maternal translocation has also been reported.[4,5]

Antenatal USG shows enlarged hyperechoic lungs, a dilated tracheobronchial tree, ascites, and an inverted or flattened diaphragm. In laryngeal atresia, the trachea is dilated because of nonclearance of fluid (which is normally secreted by the lungs). In high airway obstruction, the nonclearance of fluid from the lungs results in parenchymal hyperplasia, which is apparent on USG as enlarged hyperechoic lungs; this condition was recognized by Dolkart et al.,[6] Morrison et al.,[7] and Liggins.[8] An enlarged lung causes compression of the great veins and the right atrium, and this leads to ascites.[7,9] Compression of the esophagus due to a dilated trachea results in polyhydramnios.[10]

This malformation is generally fatal; however, there are reports of a few cases that have been successfully treated with neonatal interventions such as ex utero intrapartum treatment (EXIT).[11–12]
